# Effects of Glutathione on Growth, Intestinal Antioxidant Capacity, Histology, Gene Expression, and Microbiota of Juvenile Triploid *Oncorhynchus mykiss*

**DOI:** 10.3389/fphys.2021.784852

**Published:** 2021-11-29

**Authors:** Chang’an Wang, Baohui Su, Shaoxia Lu, Shicheng Han, Haibo Jiang, Zhuang Li, Yang Liu, Hongbai Liu, Yuhong Yang

**Affiliations:** ^1^Key Open Laboratory of Cold Water Fish Germplasm Resources and Breeding of Heilongjiang Province, Heilongjiang River Fisheries Research Institute, Chinese Academy of Fishery Sciences, Harbin, China; ^2^College of Animal Science, Northeast Agricultural University, Harbin, China; ^3^College of Animal Science, Guizhou University, Guiyang, China; ^4^Fishery Technical Extension Station of Jilin Province, Changchun, China

**Keywords:** triploid *O. mykiss*, glutathione, intestinal health, growth, microbiota

## Abstract

This study aimed to demonstrate the effects of dietary glutathione (GSH) on growth, intestinal antioxidant capacity, histology, gene expression, and microbiota in juvenile triploid rainbow trout (*Oncorhynchus mykiss*). Different diets (G0-control, G100, G200, G400, and G800) containing graded levels of GSH (0, 100, 200, 400, and 800mgkg^−1^) were fed to triplicate groups of 30 fish (initial mean weight 4.12±0.04g) for 56days. G400 had significantly improved weight gain and feed conversion rate. Based on the broken-line regression analysis, the optimum dietary GSH level was 447.06mgkg^−1^. Catalase and superoxide dismutase activities were significantly higher in G200–G800. G200 had significantly lower malondialdehyde content. The height of the intestinal muscular layer in G400 was significantly higher than that of the control group. Intestinal PepT1 and SLC1A5 gene expression was significantly increased, and the highest was observed in G400. TNF-α, IL-1β, IL-2, and IL-8 expression were significantly decreased than that of G0. Next-generation sequencing of the 16S rDNA showed a significant difference in alpha diversity whereas no differences in beta diversity. On the genus level, LefSe analysis of indicator OTUs showed *Ilumatobacter*, *Peptoniphilus*, *Limnobacter*, *Mizugakiibacter*, *Chelatococcus*, *Stella*, *Filimonas*, and *Streptosporangium* were associated with the treatment diet, whereas *Arcobacter*, *Ferrovibrio*, *Buchnera*, *Chitinophaga*, *Stenotrophobacter*, *Solimonadaceae*, *Polycyclovorans*, *Rhodococcus*, *Ramlibacter*, and *Azohydromonas* were associated with the control diet. In summary, feeding juvenile triploid *O. mykiss* 200–800mgkg^−1^ GSH improved growth and intestinal health.

## Introduction

Fish in intensive aquaculture are frequently subjected to a range of negative environmental stresses, including high temperatures, overcrowding, deteriorating water quality, pathogen invasion, and disinfectant and antibiotic (Ming et al., 2015). This causes bacterial resistance and residues in aquatic products, transfer of antimicrobial resistance genes into the environment and food, immunosuppression in fish and increased susceptibility to different diseases (Liu et al., 2010), which can lead to significant economic losses. As a consequence, one of the methods is to improve fish immune and stress resistance through dietary approaches.

Glutathione (GSH), a tripeptide consisting of glutamate, cysteine, and glycine residues (Kosower and Kosower, 1978) can eliminate unnecessary free oxygen radicals from cells. It also has many physiological roles, such as increasing antioxidant activity (Doyotte et al., 1997), protecting liver cells (Ponsoda et al., 1999), maintaining DNA synthesis, enhancing immunity (Will, 1999), and alleviating neuron intoxication (Raghunathan et al., 2007). Several GSH studies in freshwater fish and shrimp found growth, antioxidant ability, and immunity could be considerably improved in European bass (*Dicentrarchus labrax*; Zambonino-Infante et al., 1997), Nile tilapia (*Oreochromis niloticus*; Zhou et al., 2013), grass carp (*Ctenopharyngodon idella*; Ming et al., 2015), Atlantic salmon (*Salmo salar*; Ma et al., 2019), and whiteleg shrimp (*Litopenaeus vannamei*; Xia and Wu, 2018).

Rainbow trout (*Oncorhynchus mykiss*) is a typical coldwater fish. As one of China’s major farmed species, triploid *O. mykiss* is grown mainly in cold-water regions. Induced triploidy through chromosome set manipulation leads to an additional chromosome package in each somatic cell that makes fish sterile (Thorgaard, 1983). The negative effects of gonad growth with female triploid salmonids can often benefit recirculating aquaculture producers (Good and Davidson, 2016). Due to physiological changes, there may be variations in nutritional needs between ploidies (Fjelldal et al., 2016). This may be either because of increased growth potential or the odd number of chromosomes inherent in genetic variations (Ren et al., 2017). Currently, there is little information on the nutritional needs of triploid *O. mykiss*.

The intestine is a delicate tissue that plays an important role in fish health and nutrition. Maintaining intestinal homeostasis is important to enhance the growth performance and fish health status (Rombout et al. 2011). Intestinal health is determined by host (immunity, mucosal barrier), nutritional, microbial, and environmental factors (Kelly and Salinas, 2017). Thus, intestinal health could be improved through dietary approaches. GSH may benefit intestinal health in aquatic animals. In shrimp, dietary glutathione (150–250mgkg^−1^) improved the jejunum wall thickness and villus height of intestine (Wang et al., 2018). To date, there is limited published research on the intestinal health of dietary GSH in fish. Furthermore, the published data were mainly based on the purified diets. Further studies based on the practical diet are needed for the application of GSH or related ingredients in fish feeds, especially for the commercial fish feeds. Therefore, the aim of this research was to study the effects of GSH supplementation in practical diets on growth, intestinal antioxidant capacity, histology, gene expression, and microbiota in triploid *O. mykiss* diets.

## Materials and Methods

### Diets

Experimental diets (G0, G100, G200, G400, and G800) with five GSH levels (0, 100, 200, 400, and 800mgkg^−1^) were prepared (Table 1). The spectrophotometric approach (Ming et al., 2015) revealed that the dietary GSH levels were 8.52, 106.36, 210.32, 409.51, and 796.55mgkg^−1^, respectively. Fish meal, soybean protein concentrate, chicken meal, and extruded soybean were the main protein sources in the experimental diets, with fish oil and soybean oil as lipid sources and wheat middling as a carbohydrate source. Ingredients were finely ground before mixing (<250μm) and then blended with minerals and vitamins. After adding the lipid source, all the ingredients were thoroughly mixed for 15min before being mixed again for 10min. The dough was shaped into feed pellets with a diameter of 1.2mm using a small-scale extruder (G250; Machine Factory of Muyang, China). After pelleting, feed pellets were dried for approximately 12h in a ventilated oven at 45°C, then sieved. The pellets were then frozen at −20°C until use.

**Table 1:** Formulation and chemical proximate composition of the experimental diets.

Ingredients (gkg^−1^)	G0	G100	G200	G400	G800
Soybean protein concentrate^1^	300	300	300	300	300
Fish meal^2^	200	200	200	200	200
Wheat middling^3^	200	200	200	200	200
Extruded soybean^4^	145	145	145	145	145
Chicken meal^5^	50	50	50	50	50
Fish oil^6^	20	20	20	20	20
Soybean oil	30	30	30	30	30
Soybean phospholipid	30	30	30	30	30
Calcium dihydrogen phosphate	10	10	10	10	10
Vitamin premix^7^	3	3	3	3	3
Mineral premix^8^	6	6	6	6	6
GSH (mgkg^−1^)	0	100	200	400	800
Glycine	6	5.9	5.8	5.6	5.2
Proximate analysis of experimental diet					
Moisture	9.23	9.18	9.19	9.20	9.21
Crude protein	437.1	436.8	436.6	435.8	436.9
Crude lipid	109.3	108.6	109.1	109.2	109.5
Ash	6.68	6.71	6.53	6.61	6.56
Gross energy (MJkg^−1^)	18.65	18.59	18.61	18.56	18.62

1 *Dalong Feed Company, Harbin, China*.

2 *Dalong Feed Company, Harbin, China*.

3 *Huada Feed Company, Harbin, China*.

4 *Dalong Feed Company, Harbin, China*.

5 *Dalong Feed Company, Harbin, China*.

6 *Huludao Chia Tai Feed Corporation, Huludao, China*.

7 *Vitamin premix (mgkg^−1^): ascorbic acid 200, alpha-tocopherol 100, menadione sodium bisulfate 5, retinol acetate 5.2, cholecalciferol 0.07, thiamin 25, riboflavin 40, pyridoxine 25, cyanocobalamin 0.8, nicotinic acid 275, folic acid 8, biotin 5, pantothenic acid 100*.

8 *Mineral premix (mgkg^−1^): MgSO_4_·7H_2_O 2000, KCl 1,500, FeSO_4_·7H_2_O 1,000, CuSO_4_·5H_2_O 20, MnSO_4_·4H_2_O 100, ZnSO4·7H_2_O 150, KI 3, NaCl 500, CoCl_2_ 5, Na_2_SeO_3_ 3*.

### Feeding Management

Before feeding the basal diet, the fish were acclimatized in the laboratory (Chinese Academy of Fishery Sciences Coldwater Fish Experimental Station, Mudanjiang, China) for 14days. At the start of the experiment, the fish were starved for 24h before being pooled. In 15 tanks, 450 fish (initial weight 4.12±0.04g) were distributed (size: 300L). Each group had 3 tanks, and each tank was used as a replicate. The fish in each tank were weighed in batches. During the 56-day feeding trial, the fish were hand-fed four times a day (08:00, 11:00, 14:00, and 17:00) until they were satiated. Fish were raised in a water flow-through system (flow rate: 0.2Ls^−1^). Water quality was measured (YSI 6600 V2-2, Ohio State, United States) daily during the experimental period, water temperature (11.3–15.8°C), pH (7.2–7.5), dissolved oxygen (7.8–9.2mgL^−1^), and ammonia nitrogen (<0.2mgL^−1^). After a 24-h starvation period, the fish in each tank were weighed again as a batch at the end of the feeding trial.

### Sample Collection

At the end of the experiment, all fish had been starved for 24h. Ten fish were randomly selected from each tank and anesthetized (tricaine methane sulfonate MS-222, 75mgL^−1^) before weighing. The weight (precision 0.01g) and fork length (precision 0.01cm) of the fish were then determined to calculate the condition factor (*CF*). Further, the total weight of the fish in each tank was measured to calculate the weight gain rate (WGR), specific growth rate (SGR), feed conversion rate (FCR), daily feed intake (DFI). For body composition analysis, four fish were sampled from each tank and stored at −80°C. Three fish were collected from each tank and aseptically killed in an ice bath. The body surfaces of the sampled fish were washed with 70% ethanol, and the fish were dissected using sterile surgical scissors. Then their mid-intestines (located right after the pyloric ceca, 1 inch from the stomach) were collected until being tested for antioxidant enzymes, and faecal samples from the mid-intestine were collected in sterile tubes from each dietary replicated tank and stored at −80°C for microbial analysis. The research protocol was handled following the Chinese Animal Health Protection Law and the Scientific Laboratory Animal Permit Approval (Ethical Approval No. SCXK(YU)2005-0001).

### Chemical Analysis

Normal procedures were used to determine the crude protein, crude lipid, ash, moisture, and gross energy of feed and whole body (AOAC, 2012). After acid digestion using a Kjeltec system, crude protein (N×6.25) was measured using the Kjeldahl method (KDN-102C Autoanalyzer, Xianjian, China). The ether-extraction process was used to measure crude lipid with the Soxtec System (SXT-06-analyzer, Hongji, China). Moisture was measured by oven drying for 6h at 105°C. Ash was placed in a muffle furnace at 550°C for 12h. The energy content of the diet was determined using bomb calorimetry (XRY-1A, Jingmi, China).

### Intestinal Antioxidant Capacity

Intestinal samples were weighed and mixed with an ice-cold buffer in a 1:9 ratio (0.86% normal saline). The extract was then centrifuged for 15min at 7,700*g* and 4°C, and the supernatant was used to determine superoxide dismutase (SOD), and catalase (CAT), glutathione reductase (GR) activities, GSH, and malondialdehyde (MDA) content. The standard was bovine serum albumin, and the spectrophotometer absorbance was determined at 750nm. Spectrophotometric kits were bought from the Chinese Nanjing Jiancheng Institute of Bioengineering and were used to analyze the MDA content (cat. no. A003-1), GSH content (cat. no. A006-1-1), and the activity of GR (cat. no. A062-1-1), SOD (cat. no. A001-1), and CAT (cat. no. A007-1). The concentrations of MDA, SOD, CAT, GR and GSH were then calculated according to the instructions provided with the respective kits as described by Deng et al. (2015).

### Intestinal Gene Expression

Total RNA was extracted from mid-intestines according to the manufacturer’s instructions using RNAiso Plus Reagent (TaKaRa, Dalian, China). A spectrophotometer was used to examine the absorbance at 260nm to determine the RNA concentration. Using agarose gel electrophoresis, the RNA integrity was determined, and the absorbance ratio at A260 nm/A280 nm ranged from 1.8 to 2.0. Tumor necrosis factor (TNF-α), interleukin 1 (IL-1β), interleukin 2 (IL-2), interleukin 8 (IL-8), solute carrier family 1 member 5 (SLC1A5), peptide transporter 1 (Pep T1), and β-actin expression levels were determined using quantitative real-time PCR (ABI 7500, USA) with a reaction length of 20μl, including 10μl of 2 SYBR® Premix Ex Taq (TaKaRa, Dalian, China), 0.8μl for quantitative real-time PCR, specific primers were constructed based on sequences cloned and published in the *O. mykiss* gene bank (Table 2). The cycling conditions were 95°C for 30s followed by 35cycles of 95°C for 5s, 59°C for 10s, and 72°C for 30s. The housekeeping gene (β-actin) was chosen as a reference gene to normalize the results. 2^−ΔΔCt^ was used to measure the expression values.

**Table 2:** Primers sequence and annealing temperature in RT-PCR.

Target genes	Forward primer (5ʹ-3ʹ)	Reverse primer (5ʹ-3ʹ)	Accession number
β-Actin	F: GGACTTTGAGCAGGAGATGG	R:ATGATGGAGTTGTAGGTGGTCT	XM_042314795.1
SLC1A5	F:CCTGTCAATCAACGCTGGT	R:CACTGCCCATAATGAACACG	KY775396.1
PepT1	F: CTGGGAGAGGAGGGAGAGAT	R: TCCACGATCTTCCCTGCTAC	XM_014213484.1
IL-1β	F:ACATTGCCAACCTCATCATC	R:GTTCTTCCACAGCACTCTCC	LR584424.1
IL-2	F:TGATGTAGAGGATAGTTGCATTGTTGC	R:GAAGTGTCCGTTGTGCTGTTCTC	NM_001164065.2
IL-8	F:CACAGACAGAGAAGGAAGGAAAG	R:TGCTCATCTTGGGGTTACAGA	AY160981.1
TNF-α	F:GTTGGCTATGGAGGCTGTGT	R:ACCCTCTAAATGGATGGCTG	NM_001124357.1

### Intestine Histology

The intestinal samples were fixed in Bouin’s solution for 24h, rinsed multiple times with water to extract the fixative, dehydrated in ethanol, placed in xylene, embedded in wax for 2h at 60°C, sectioned to 6μm thick, with a microtome (Leica-RM2235), stained with hematoxylin–eosin, and sealed with neutral resin. Intestinal morphology was measured using Motic Images Plus 2.0 software after being photographed (Nikon, DS-Ri2). The muscular layer and villus height were determined by randomly selecting 10 villi per slide.

### Intestine Bacterial DNA Extraction and Sequencing

Complete DNA was extracted from 0.2g of intestinal faeces using a DNA Extraction Kit (Beijing Tiangen Biochemical Technology Co. Ltd., China) according to the guidelines for total DNA extraction from intestinal microorganisms. DNA was isolated from the collected intestinal microorganisms using specific primers of the forward sequence 341F (5′-CCTAYGGGRBGCASCAG-3′) and reverse sequence 806R (5′-GGACTACNNGGGTATCTAAT-3′; Sun et al., 2020). The optimized conditions for amplification were as follows: one pre-denaturation cycle at 95°C for 5min, 27cycles of denaturation at 95°C for 30s, annealing at 55°C for 30s, elongation at 72°C for 45s, and a final extension at 72°C for 10min. The resulting amplicons were purified from 0.7% agarose gel, measured concentration using Qubit dsDNA broad-range assay kit (Life Technologies, United States), and equal concentration (20ngμl^−1^) of amplicons were pooled together, and sequenced on the Illumina MiSeq platform (300bp paired-end reads; Beijing Baimaike Technology Co. Ltd., China).

### Intestinal Microbiota Bioinformatics Analysis

Using QIIME (Version 1.8.0, http://qiime.org/), low-quality reads with quality scores <20e were filtered out and raw reads were sorted according to their Barcode sequences (Caporaso et al., 2010). The reads were first cut in poor quality and then separated based on Barcode from the subsequent reads. Through binding the raw reads to the Barcode, the raw reads were then separated from the subsequent reads. To detect chimera, the Barcode and primer sequences were removed from the quality control, and the read sequences were removed using VSearch software (https://github.com/torognes/vsearch/; Li et al., 2015). The chimeric sequences were compared to the species annotation database, and the remaining chimeric sequences were deleted. The chimeric sequences were deleted from the database, leaving only the clean reads. The final correct reads were collected using the Uparse program (Version 7.0.1001, http://www.drive5.com/uparse/) to group all clean reads from all samples (Haas et al., 2011). The sequences were grouped into operative taxonomic units (OTUs) with 97% sequence similarity. The most frequent sequences were chosen as OTUs based on the algorithm (Edgar, 2010). According to the algorithm, the most commonly occurring sequences were chosen as symbolic OTUs. The Mothur system was used to species-annotate the OTUs sequences. Taxon resembling chloroplasts, mitochondria, unknowns, archaea, and eukaryotes were removed. The RDP Classifier Bayesian algorithm (http://sourceforge.net/projects/rdpclassifier/; Wang et al., 2007) was used to cluster the samples into OTUs, as was the GreenGene database^1^ for species annotation analysis, and OTUs for abundance. Raw reads were saved to the National Center for Biotechnology Information’s (NCBI) Sequence Read Archive (SRA) database (Accession Number: PRJNA714809). At each taxonomic rank, the species abundance, diversity index (Chao1 index, Shannon index and ACE index), and population structure were studied (Lozupone and Knight, 2005).

### Calculations and Statistical Analysis

Weight gain rate (WGR, %)=100×(weight gain, g)/(initial weight, g); Survival rate (%)=100×(final number of fish)/(initial number of fish); Feed conversion ratio (FCR)=(dry dietary intake, g)/(weight gain, g). Specific growth rate (SGR, % per day)=100×[Ln (final weight, g)−Ln (initial weight, g)]/duration (days); Condition factor (CF)=100×(body weight, g)/(body length, cm)^3^; survival rate (SR, %)=100×(final fish number/initial fish number); Daily feed intake (DFI)=(feed consumed, g)/[(initial weight+final weight)/2, g]×(days, d).

All data in tables and figures are expressed as mean±standard deviation (SD). After normality and homogeneity checking, one-way variance analysis (ANOVA) and Duncan’s multiple range tests were used to examine the data. *p* values <0.05 were considered significantly different. The SPSS statistical package 23.0 (SPSS Inc., Chicago, IL, USA) was used for statistical analysis. The sigma plot software version 14.0 is used to draw column graphs and curves.

## Results

### Growth Performance

**Figure 1:**
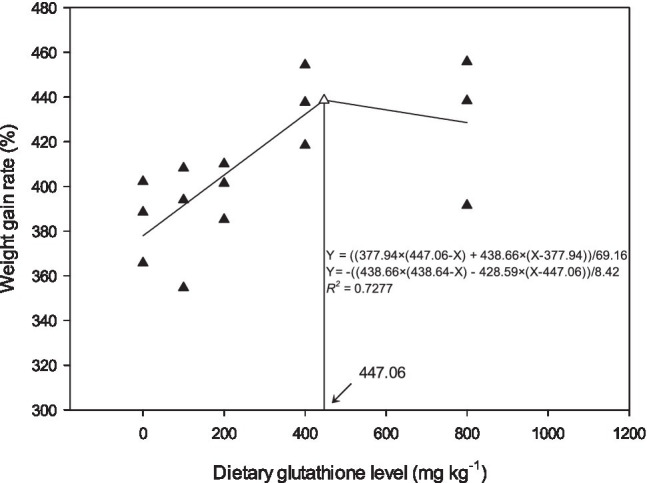
Relationship between weight gain and dietary GSH for *O. mykiss* as described by a broken line regression (*n*=3). The breakpoint in the broken line is 447.06mgkg^−1^.

The effects of dietary GSH level on weight gain, feed conversion rate, and survival rate are shown in Table 3. With increasing dietary GSH level, weight gain rate and specific growth rate gradually increased, reaching the highest level in G400, showing a significant difference with G0 and G100 (*p*<0.05). The daily feed intake of G200 and G400 was significantly higher than that of the control group (*p*<0.05). The feed conversion rate decreased with increasing GSH, and G400 was the lowest, which was significantly different from G0 and G100 (*p*<0.05). The survival rate of G400 was the highest, and significantly higher than that of G0, G100, and G200 (*p*<0.05). There were no significant differences in condition factor among the groups (*p*>0.05). According to the relationship between weight gain and dietary GSH levels, the broken line model was selected as a good fit for the model, and the optimal dietary GSH level was 447.06mgkg^−1^ (Figure 1).

**Table 3:** Growth performances of *O. mykiss* fed the experimental diets (mean±SD, *n*=3).

Indices	G0	G100	G200	G400	G800	*p*-values
IBW^1^ (g)	4.10 ± 0.12	4.13 ± 0.16	4.15 ± 0.13	4.11 ± 0.13	4.12 ± 0.17	0.991
FBW^2^ (g)	19.89 ± 0.35^a^	20.04 ± 0.40^a^	20.71 ± 0.16^ab^	22.05 ± 1.00^c^	21.74 ± 0.64^bc^	0.003
WGR^3^ (%)	385.45 ± 18.42^a^	385.62 ± 27.76^a^	398.93 ± 12.64^ab^	436.83 ± 17.97^b^	428.59 ± 33.24^ab^	0.049
SGR^4^ (% d^−1^)	2.82 ± 0.07^a^	2.82 ± 0.10^a^	2.87 ± 0.05^ab^	3.00 ± 0.06^b^	2.97 ± 0.12^ab^	0.052
FCR^5^	1.02 ± 0.06^b^	1.01 ± 0.04^b^	0.97 ± 0.02^ab^	0.92 ± 0.01^a^	0.98 ± 0.04^ab^	0.083
DFI^6^ (g d^−1^)	1.44 ± 0.02^a^	1.46 ± 0.03^ab^	1.51 ± 0.01^b^	1.51 ± 0.06^b^	1.50 ± 0.03^ab^	0.035
CF^7^	1.27 ± 0.01	1.28 ± 0.01	1.30 ± 0.03	1.30 ± 0.03	1.29 ± 0.03	0.659
Survival (%)	90.67 ± 3.06^a^	92.67 ± 3.06^ab^	92.00 ± 2.00^ab^	98.00 ± 2.00^c^	95.33 ± 1.15^bc^	0.023

*Means in the same row with different superscripts are significantly different (p<0.05)*.

1 *IBW, initial body weight*.

2 *FBW, final body weight*.

3 *WGR, weight gain rate*.

4 *SGR, specific growth rate*.

5 *FCR, feed conversion rate*.

6 *DFI, daily feed intake*.

7 *CF, condition factor*.

### Body Composition

Crude protein, crude lipid, ash, and moisture levels did not change significantly among the groups (*p*>0.05; Table 4).

**Table 4:** Effects of dietary GSH on body composition of *O. mykiss* (mean±SD, *n*=12).

Groups	Moisture (%)	Crude protein (%)	Crude lipid (%)	Ash (%)
G0	74.76 ± 1.05	18.48 ± 0.72	7.25 ± 0.64	2.21 ± 0.13
G100	74.47 ± 0.70	17.82 ± 0.17	6.61 ± 0.95	2.06 ± 0.08
G200	74.25 ± 0.77	18.13 ± 0.47	7.35 ± 0.41	2.28 ± 0.16
G400	73.84 ± 0.66	17.68 ± 0.71	6.59 ± 0.52	2.05 ± 0.08
G800	73.96 ± 0.11	17.93 ± 0.40	7.00 ± 0.72	2.17 ± 0.15
*P*-values	0.555	0.448	0.536	0.206

### Antioxidant Capacity

GSH content and GR activity did not vary considerably with increased dietary GSH supplementation (Table 5). Compared to the control group, SOD and CAT activities were significantly higher in G200, G400, and G800 (*p*<0.05). MDA content was significantly lower in G200 than in the other groups (*p*<0.05).

**Table 5:** Effects of dietary GSH on the antioxidant capacity of mid-intestine in *O. mykiss* (mean±SD, *n*=9).

Groups	SOD/(U mg^−1^ protein)	CAT/(Umg^−1^ protein)	GR/(Umg^−1^ protein)	GSH/(μmolg^−1^ protein)	MDA/(nmolg^−1^ protein)
G0	303.60 ± 16.80^a^	306.33 ± 12.50^a^	46.94 ± 2.64	37.88 ± 5.28	0.95 ± 0.03^c^
G100	320.40 ± 4.33^a^	328.33 ± 30.35^ab^	46.04 ± 1.54	36.08 ± 3.09	0.90 ± 0.01^bc^
G200	384.40 ± 33.13^b^	391.67 ± 14.22^d^	46.20 ± 1.74	36.40 ± 3.48	0.77 ± 0.11^a^
G400	382.80 ± 5.23^b^	368.33 ± 3.51^cd^	47.44 ± 0.21	38.88 ± 0.42	0.83 ± 0.03^ab^
G800	361.20 ± 14.55^b^	349.00 ± 26.00^bc^	46.02 ± 0.98	36.04 ± 1.96	0.87 ± 0.01^bc^
*P*-values	0.001	0.003	0.771	0.770	0.018

*The superscript small letters in the same column mean the significant difference at p<0.05*.

## Intestine Histology

**Figure 2:**
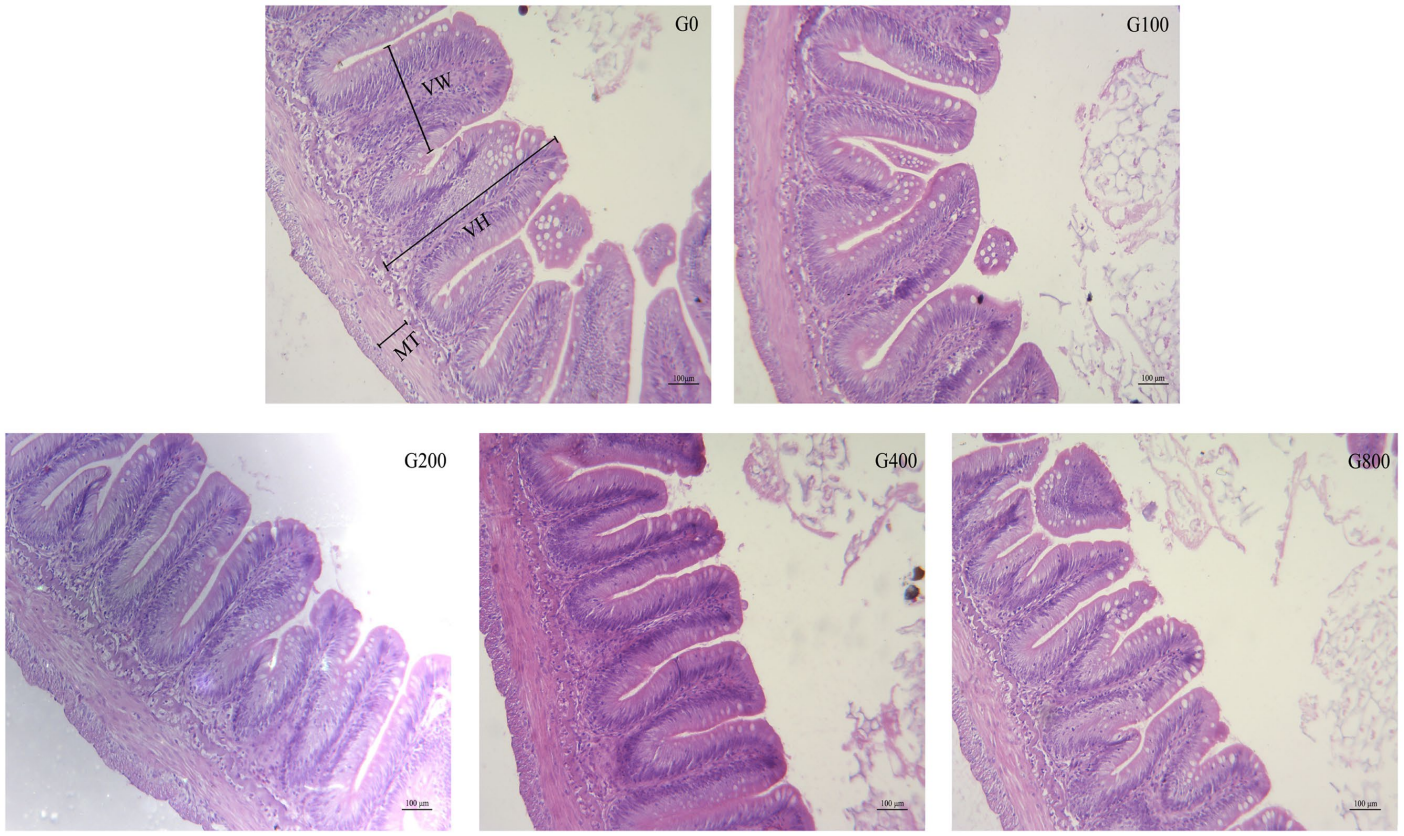
The light micrograph in the triploid *O. mykiss* fed GSH diets. VH, VW and MT represent villus height, villus width and muscular thickness.

Table 6 and Figure 2 show the mid-intestine morphology. No pathological differences in the intestines were found between the different groups. When the dietary GSH level was 0–200mgkg^−1^, the height of intestinal muscular layer was not significantly different (*p*>0.05); however, it was significantly higher in G400 (*p*<0.05). The intestinal villus height and width were also not significantly different between groups (*p*>0.05).

**Table 6:** Effects of dietary GSH on micro-morphology of the intestine of *O. mykiss* (mean±SD, *n*=9).

Groups	muscular layer (μm)	villus height (μm)	villus width (μm)
G0	120.94 ± 8.95^a^	626.04 ± 36.51	208.06 ± 16.52
G100	128.70 ± 4.67^ab^	634.04 ± 33.48	214.78 ± 21.11
G200	131.04 ± 11.87^ab^	636.96 ± 87.82	219.98 ± 36.43
G400	142.16 ± 9.44^b^	685.73 ± 12.11	234.90 ± 53.65
G800	137.58 ± 10.91^ab^	655.93 ± 50.01	232.47 ± 45.83
*P*-values	0.020	0.628	0.884

*The superscript small letters in the same column mean the significant difference at p<0.05*.

### Gene Expression

**Figure 3:**
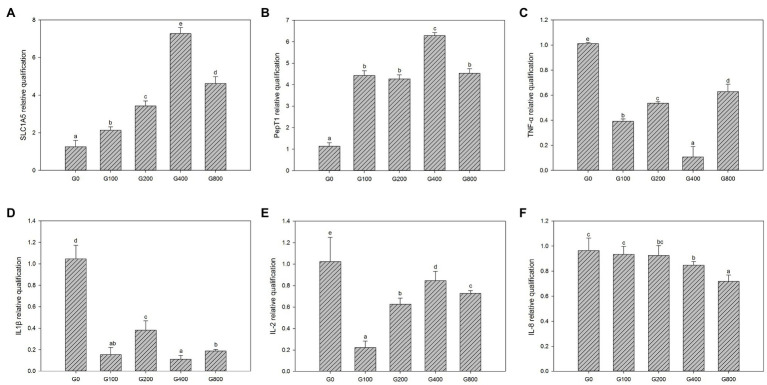
The gene expression of the intestine in each group (mean±SD, *n*=3). **(A)** SLC1A5, **(B)** pepT1, **(C)** TNF-α, **(D)** IL-1β, **(E)** IL-2, and **(F)** IL-8 [β-actin was chosen as a reference gene to normalize the results, different letters indicate significant differences between the different groups (*p*<0.05)].

Figure 3 shows the mid-intestine gene expression. Compared to that of the control group, PepT1 and SLC1A5 gene expression in each GSH feeding group were significantly increased (*p*<0.05); G400 had the highest expression, and there was no significant difference between G100, G200, and G800 (*p*>0.05). TNF-α, IL-1β, IL-2, and IL-8 gene expressions were significantly reduced than that of the control group, respectively.

### Microbial Diversity

**Figure 4:**
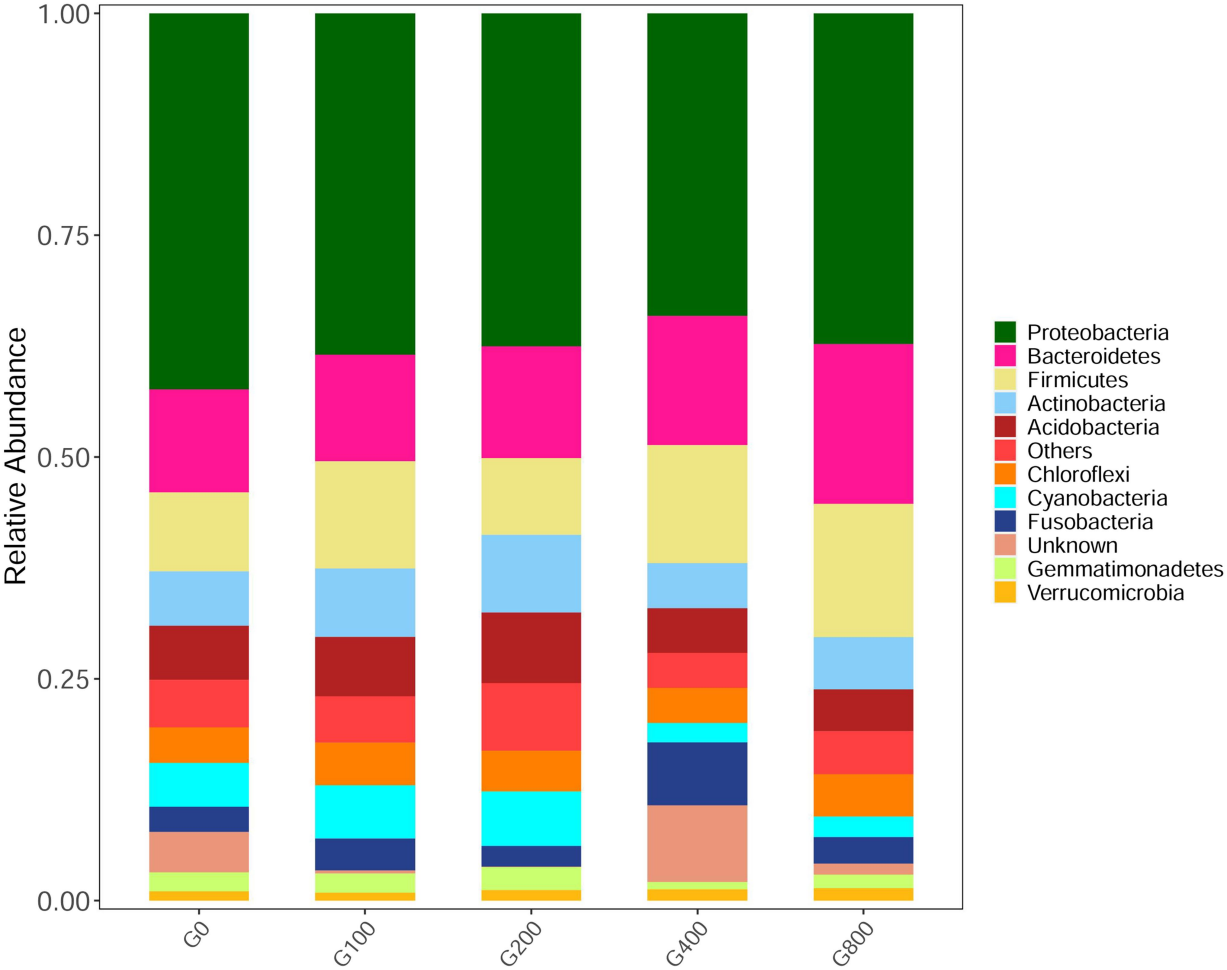
Composition and relative abundance of bacterial community-based 16S rDNA sequences in phylum level (*n*=3).

**Figure 5:**
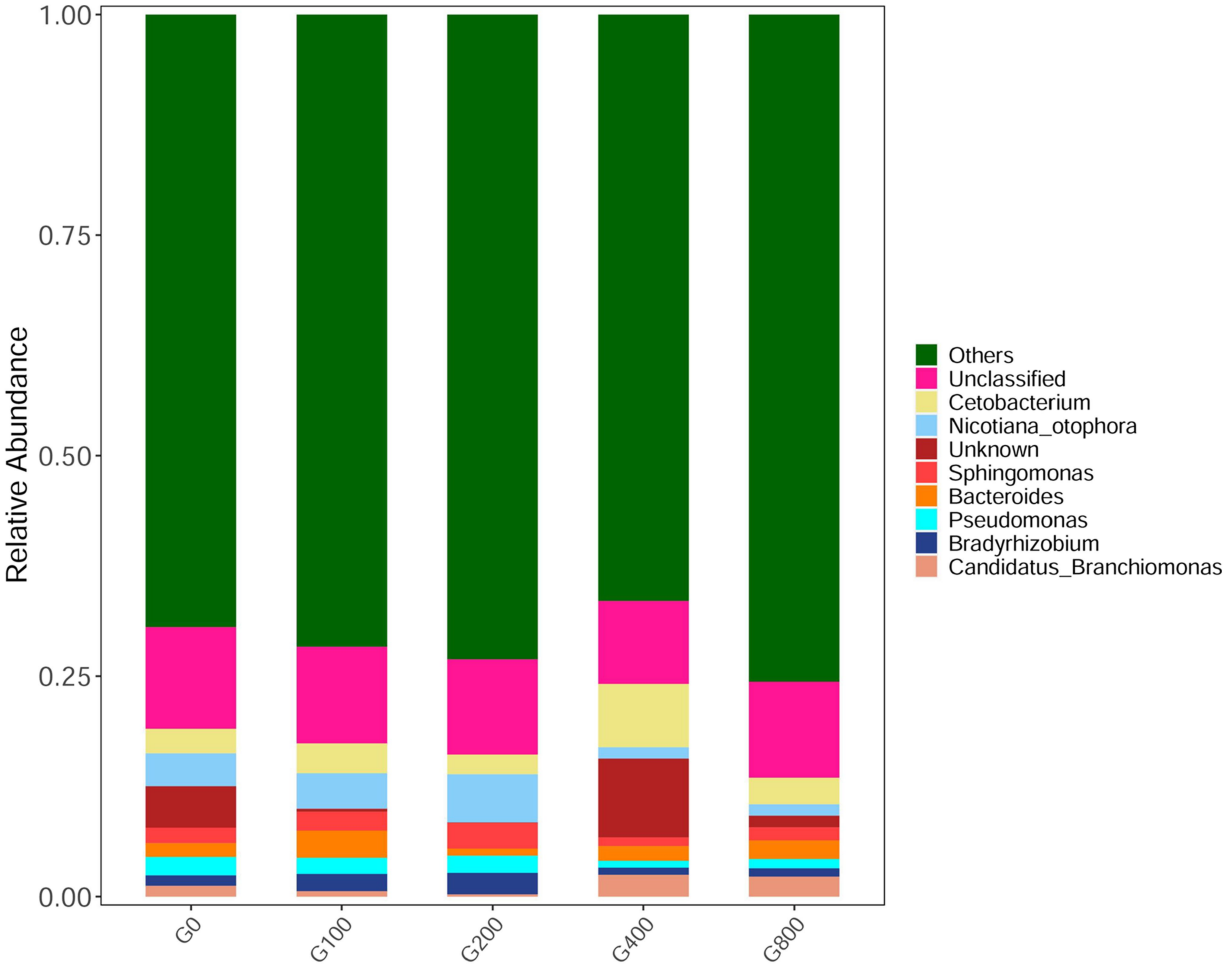
Composition and relative abundance of bacterial community-based 16S rDNA sequences in genus level (*n*=3).

**Figure 6:**
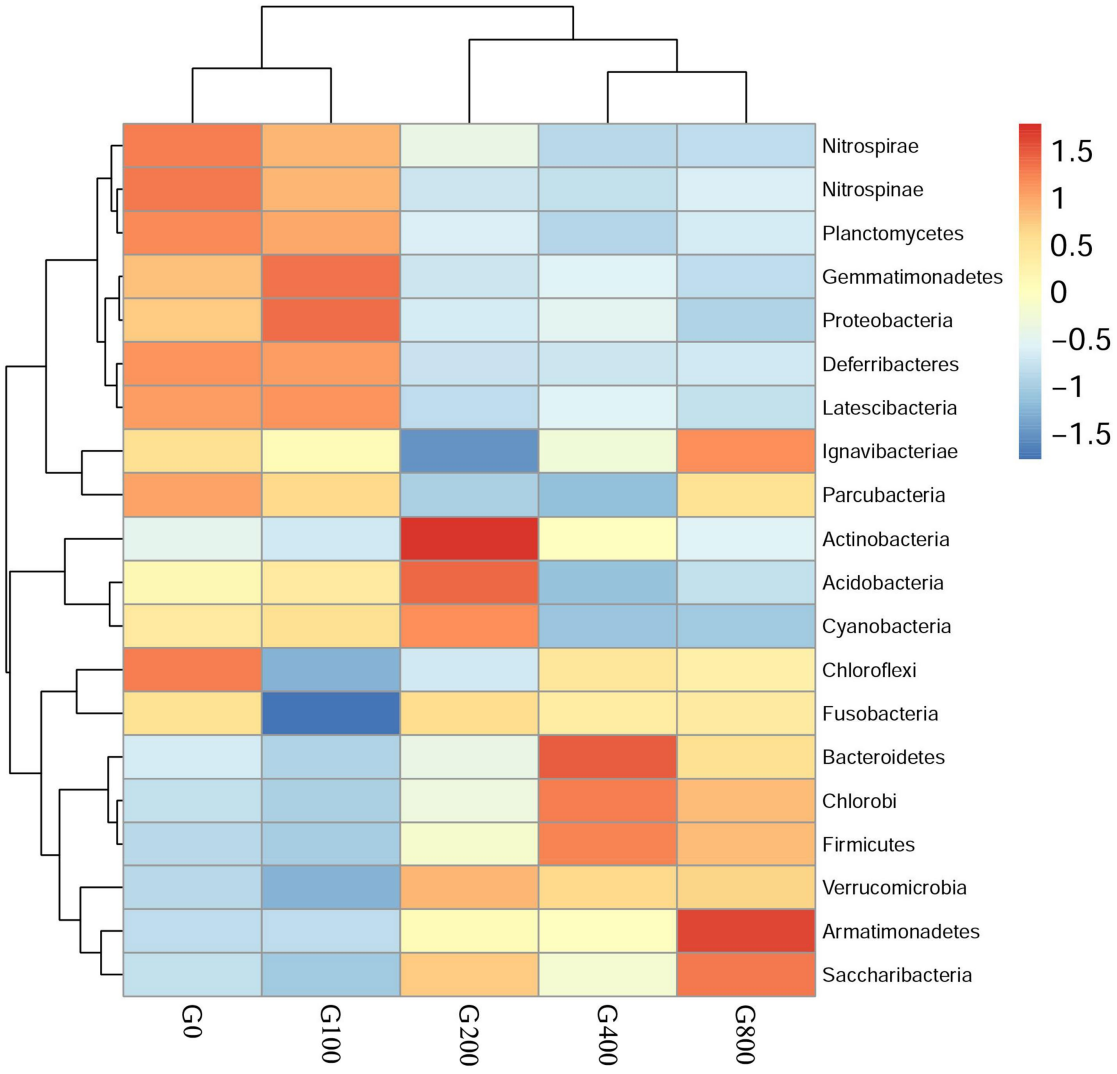
Heatmap analysis of the species abundance clustering in the top 20 on the phylum level (mean±SD, *n*=3).

The 16S rDNA sequencing produced 1.18 million reads with a mean of 68,310 clean tags per sample. Chloroplast, mitochondria and eukaryotes were removed and reduced the amount of reads by 66.7% to a total of 1.0 million reads. Table 7 shows the Chao1, ACE, and Shannon indexes. Of all experimental treatments, fish fed a G400 diet had the highest values of Chao1, ACE, and Shannon index. On the phylum level, dietary GSH resulted in reduced Proteobacteria abundance and increased Bacteroidetes, Firmicutes, Acideobacteria, and Actinobacteria abundance (Figure 4). On the genus level, faeces were mainly composed of *Cetobacterium*, *Nicotiana_otophora*, *Sphingomonas*, *Bacteroides*, *Pseudomonas*, *Bradyrhizobium*, and *Candidatus_Branchiomona* (Figure 5). Figure 6 depicts a heatmap study of species abundance clustering at the phylum stage. Clustering results showed that the intestinal microbiota composition was classified into three classes (G0, G100; G400, G800; G200).

**Table 7:** Effects of dietary GSH on alpha diversity index of intestinal microbiota in *O. mykiss* (mean±SD, *n*=3).

Groups	OTU	ACE index	Chao1 index	Shannon index	Coverage(%)
G0	1282.67 ± 658.73	1222.18±91.65^a^	1052.81±77.73^a^	6.05±0.04^a^	99.88
G100	1069.67 ± 189.83	1213.19±48.52^a^	1246.05±49.31^a^	6.12±0.13^ab^	99.85
G200	2572.00 ± 1587.76	3978.54±208.12^bc^	4007.93±216.42^cb^	6.76±0.35^c^	98.84
G400	2881.00 ± 1317.55	4211.87±233.62^c^	4304.58±205.83^c^	6.62±0.22^c^	98.44
G800	2431.33 ± 1022.59	3060.39±137.16^b^	3139.11±196.50^b^	6.47±0.03^bc^	99.41
*p*-values	0.485	0.032	0.021	0.043	—

*The superscript small letters in the same column mean the significant difference at p<0.05*.

**Figure 7:**
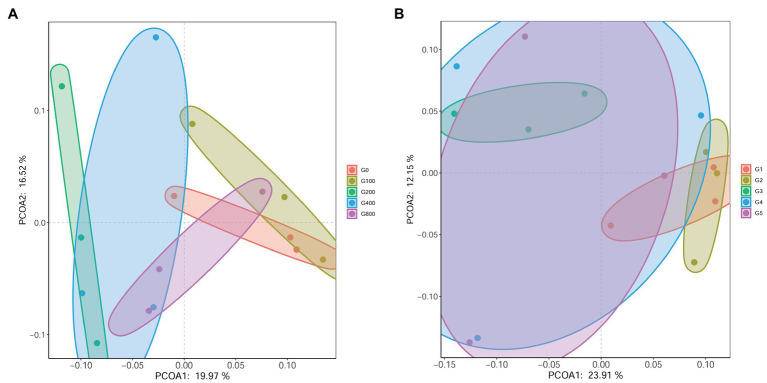
Principle coordinate analysis (PCoA) based on weighted-unifrac **(A)** and unweighted-unifrac **(B)** analysis of bacterial profiles from intestines of *O. mykiss* (*n*=3).

Principle coordinate analysis (PCoA) illustrated beta diversity. Samples in PCoA using weighted and unweighted UniFrac distances were evenly distributed among the groups (Figure 7). The findings revealed that the intestinal microbiota composition of G0 and G100 are similar. However, there was no significant difference in beta diversity using ANOSIM (*R*=0.049, *p*=0.328).

Analysis with LefSe noted several indicator bacteria species associated with each diet (Table 8). In the faeces, dietary GSH 200–800mgkg^−1^ increased the abundance of *Ilumatobacter*, *Peptoniphilus*, *Limnobacter*, *Mizugakiibacter*, *Chelatococcus*, *Stella*, *Filimonas*, and *Streptosporangium*, while the control diet had increased abundance of *Ferrovibrio*, *Buchnera*, *Chitinophaga*, *Stenotrophobacter*, *Solimonadaceae*, *Polycyclovorans*, *Rhodococcus*, *Ramlibacter*, *Azohydromonas*, and *Arcobacter*.

**Table 8:** Linear discriminant analysis effect size (Lefse) of indicator bacteria species that were significantly (*p*<0.05) associated with each group.

Groups	Phylum	Family/Genus	LDA	*p*-values
G0	Proteobacteria	*Buchnera*	2.712	0.028
G0	Bacteroidetes	*Chitinophaga*	2.696	0.040
G0	Acidobacteria	*Stenotrophobacter*	2.318	0.019
G0	Proteobacteria	Solimonadaceae	2.312	0.035
G0	Proteobacteria	*Polycyclovorans*	2.259	0.032
G0	Actinobacteria	*Rhodococcus*	2.230	0.035
G0	Proteobacteria	*Ramlibacter*	2.193	0.039
G0	Proteobacteria	*Ferrovibrio*	2.100	0.014
G0	Proteobacteria	*Azohydromonas*	2.056	0.026
G0	Proteobacteria	*Arcobacter*	2.008	0.045
G200	Actinobacteria	*Streptosporangium*	2.021	0.029
G200	Bacteroidetes	*Filimonas*	2.023	0.039
G400	Actinobacteria	*Ilumatobacter*	2.763	0.029
G400	Proteobacteria	*Mizugakiibacter*	2.265	0.034
G400	Proteobacteria	*Chelatococcus*	2.235	0.042
G400	Proteobacteria	*Stella*	2.161	0.039
G800	Proteobacteria	Rhodobacteraceae	3.514	0.042
G800	Firmicutes	*Peptoniphilus*	2.511	0.041
G800	Proteobacteria	*Limnobacter*	2.365	0.043

### Microbial Function

**Figure 8:**
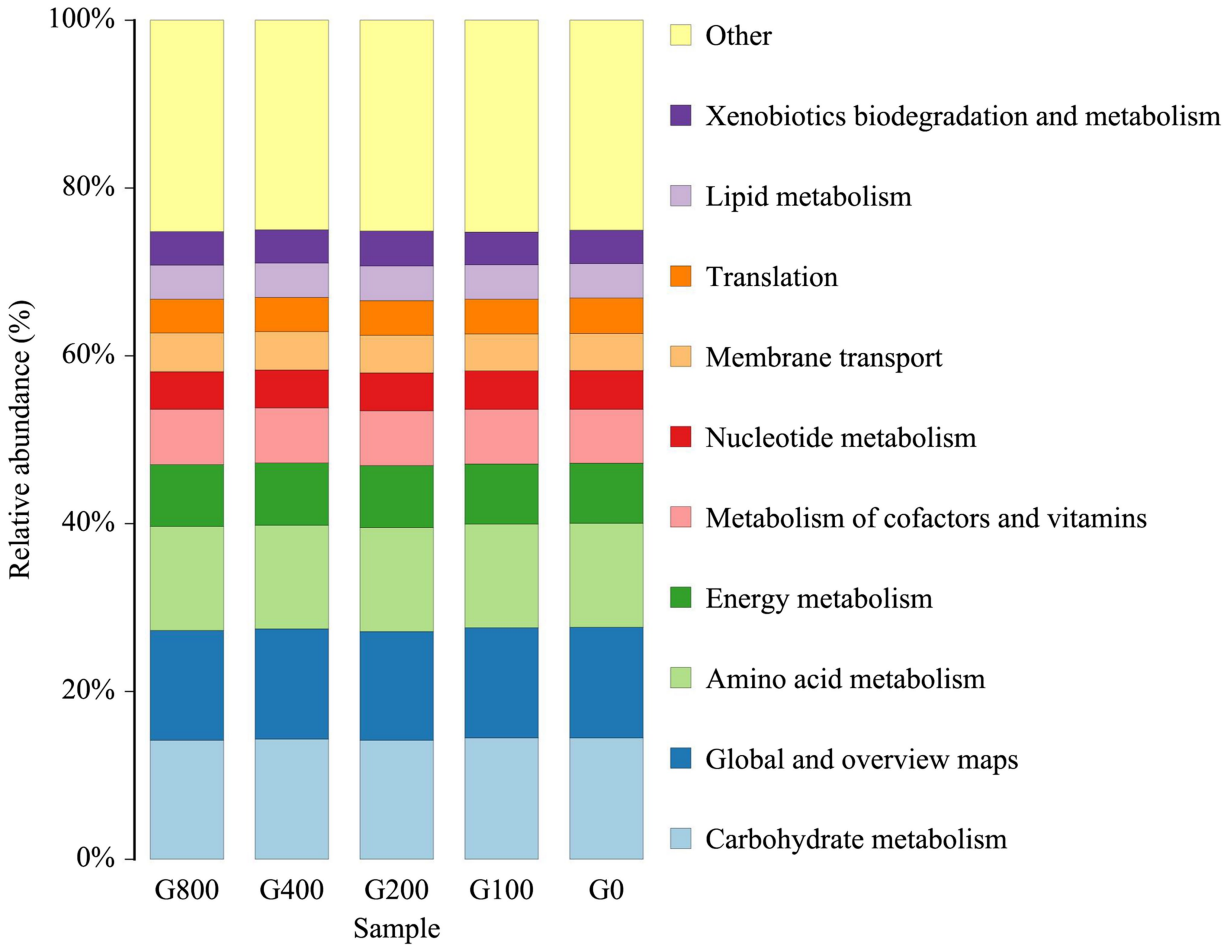
Microbial functions in the top 10 of level-2 for intestine samples (mean±SD, *n*=3).

The microbial functions of the intestine were predicted with PICRUSt2 (Phylogenetic Investigation of Communities by Reconstruction of Unobserved States; Figure 8). The intestine microbiota was enriched with functions related to carbohydrate metabolism, amino acid metabolism, energy metabolism, the metabolism of cofactors and vitamins, nucleotide metabolism, membrane transport, translation, lipid metabolism, and xenobiotics biodegradation and metabolism. However, the abundant among the groups had little change, and it is speculated that the microbial functions of the intestine were similar.

## Discussion

### Growth Performance

The results showed that dietary GSH can significantly improve the weight gain rate and survival rate of triploid *O. mykiss*, and reduce the feed conversion rate, which is similar to results obtained for grass carp (Ming et al., 2015) and Atlantic salmon (Ma et al., 2019). GSH can enhance the growth performance of European bass larvae by improving amylase and protease activity (Zambonino-Infante et al., 1997). In a study of Japanese flounder (*Paralichthys olivaceus*), the deamination product of cysteine and hydrolysate of GSH was found to be a component of coenzyme A (Wang et al., 2011). This can also enhance grass carp growth by destroying the growth inhibitory molecules, and promoting the secretion of growth hormone (Xiao and Lin, 2003). It was found that GSH can promote the secretion of pituitary growth hormone and liver insulin-like growth factor-1 level, to promote the synthesis of protein and improve the nutrient utilization rate (Ming et al., 2015). Furthermore, GSH can protect intestinal mucosa from the damage of toxins and peroxides, protect the digestive system, and help the intestinal absorption of nutrients in animals (Aw et al., 1992), which is in line with reports of improving growth and feed conversion rate.

In the present study, the optimal addition of GSH in the feed of triploid *O. mykiss* was 447.06mgkg^−1^, which is close to that of grass carp (381mgkg^−1^; Ming et al., 2015), but much higher than that of Japanese flounder (368.92mg·kg^−1^; Wang et al., 2011) and Nile tilapia (355.13mgkg^−1^; Zhou et al., 2013). The differences in these results may be related to the culture environment, species and size, feed formula, feeding strategy, and raw material processing technology. Adding an appropriate amount of GSH to the feed can improve the growth performance, but excessive GSH will result in negative effects. The weight and protein efficiency of yellow catfish (*Pelteobagrus fulvidraco*) first increased and then decreased, and reached a maximum value at 300mgkg^−1^, and the high GSH levels possibly had a toxic effect on the fish (Zhou et al., 2017). A low GSH concentration can inhibit lipid peroxidation, whereas a high GSH concentration can promote mitochondrial lipid peroxidation, which is related to the mutual transformation of reduced glutathione and oxidized glutathione in the body (Gao et al., 1998). Moreover, GSH can be synthesized with many compounds, such as aromatic epoxides, halogenated hydrocarbons, to produce toxic metabolites, which cause DNA damage and have adverse effects on the body (Kleider et al., 2016).

### Body Composition

The effects of GSH on fish body composition varied with different species. There was no significantly different effect of GSH on the dry matter, crude lipid, and ash content of grass carp (Ming et al., 2015). Similarly, there were no significant differences among the groups for body composition in this study. However, crude lipid and protein in yellow catfish were higher than that of the control group (Zhou et al., 2017). This may be related to GSH participating in the transport of amino acids and small peptides, which promotes amino acid absorption (Meister and Anderson, 1983).

### Histology

Increased intestinal villus height can enhance digestion and absorption by improving the interaction between the intestine and nutrients (Long et al., 2018). The results in this study were similar to previous reports of Atlantic salmon (Refstie et al., 2000) and meager (*Argyrosomus regius*; Ribeiro et al., 2015). The small peptide can improve the intestinal development of fish, and increase the height of small intestinal villi (Murashita et al., 2015). It has been reported that adding GSH to the feed of Pacific white shrimp improved intestinal development, promoted intestinal villi growth, and thickened the muscle layer (Wang et al., 2018). It was found that when fish were fed dietary GSH at 400mgkg^−1^, the myometrium thickness and intestinal villus height increased significantly. The results showed that GSH could enhance intestinal tract development by affecting the tissue structure of the small intestine, improve the utilization rate of feed nutrition, and enhance the growth and development of triploid *O. mykiss*.

### Antioxidant Capacity

In the present study, the SOD, CAT, GSH, and glutathione reductase (GR) activities in the mid-intestine when fish fed GSH were higher than those of the control group. Similarly, the SOD activity also increased as dietary GSH levels increased (Zhou et al., 2012). It has been speculated that exogenous GSH can reduce the oxygen-free radical damage to the body and antioxidant stress of *O. mykiss* (Pena-Llopis et al., 2001). However, the SOD activity of Japanese flounder was not affected by the amount of GSH added to the feed (Wang et al., 2011). It is possible that the effect of GSH on SOD activity is different for different species. With the increase of GSH, CAT activity in tilapia increased first and then decreased; therefore, CAT could possibly eliminate hydrogen peroxide in the body and protect cells from its toxicity (Zhou et al., 2012). The change in CAT activity was due to the activation of the antioxidant enzyme system in the body. In this study, the mid-intestine CAT activity in triploid *O. mykiss* was higher than that of the control group. Therefore, adding appropriate GSH could regulate body antioxidant status, decrease the concentration of hydrogen peroxide in the cells, and reduce the degree of cell damage. In this experiment, the mid-intestine GSH and GR activities in triploid *O. mykiss* are higher than those in the control group. It is speculated that exogenous GSH could activate a reaction and metabolism related to GSH and increase the demand for GSH. Therefore, the GR activity that can convert oxidized GSH into GSH (Ming et al., 2015) also increased accordingly. Furthermore, intestinal MDA content when fish were fed GSH was lower than that of the control group, which is associated with previous study findings (Wang et al., 2011). It is possible that an appropriate amount of GSH could reduce the toxic effect of lipid peroxidation in cells, reducing the degree of cell damage (Birnie-Gauvin et al., 2017).

### Gene Expression

When protein ingestion increases, the PepT1 expression in the brush border of the small intestine increases, transport efficiency of small peptides increases, and the use of protein, tripeptides, and free amino acids will be improved through enhanced amino acid uptake efficiency (Ostaszewska et al., 2010). SLC1A5, an amino acid transporter, transports amino acids using the concentration gradient of Na^+^ inside and outside the cell membrane and is the most important transporter for cells to absorb exogenous glutamine. Compared to that of the control group, the PepT1 and SLC1A5 gene expression in the intestinal oligopeptide transporter by dietary GSH supplementation increased significantly, suggesting that dietary glutamine utilization was improved, which enhances intestinal development.

Proinflammatory factors are necessary to signal cytokines to initiate and regulate this reaction, which mainly includes tumor necrosis factor-α, interleukin-1β, interleukin-2, and interleukin-8. Interleukin-1β can promote the proliferation and activation of immune cells, such as thymocytes and T cells, and also promote the synthesis and secretion of immune proteins by B cells, and mediate the inflammatory response (Sims and Smith, 2010). Interleukin-2 is primarily generated by activated T cells, which can promote the growth, proliferation, and differentiation of lymphocytes, enhance NK cell function and play an important role in immune response and antiviral infection (Koreth et al., 2011). Interleukin-8 regulates immunity, promoting cell mitosis, and stimulating capillary formation (Hoffmann et al., 2002). Tumor necrosis factor-α is a member of the cytokine family, which can induce apoptosis of tumor cells and coordinate non-specific immune response (Paul, 1985), which is produced in the early stage of inflammatory response. When the level of intestinal pro-inflammatory factors increases, it promotes the production of the inflammatory response, causing damage to intestinal mucosa tissue cells, destroying the barrier function of the intestinal epithelium, increasing intestinal permeability, making pathogens and endotoxins enter the blood circulation, causing functional damage to more organs, resulting in a systemic inflammatory response of the body (Costantini et al., 2010). It has been found that GSH can reduce intestinal injury, the expression of intestinal inflammatory factors, incidence of pathogen and endotoxin translocation in the intestinal tract of rats with acute necrotizing pancreatitis, and play a role in protecting the intestinal mucosa (Aw et al., 1992). In addition to interleukin-8, the expression of proinflammatory factors in the intestinal tract in the treatment group was significantly lower than that of the other groups. The mechanism of action may be related to the NF-κB/MLCK pathway inhibition by GSH’s nitroso derivatives, to protect the structure and function of closely connected intestinal epithelial cells (Koeberle et al., 2020).

### Intestinal Microbiota

Feed composition is a key aspect influencing fish intestinal microbiota (Ringø et al., 2006). In this study, when fish were fed GSH, the Chao1 index, ACE index, and Shannon index increased, which indicated that the abundance and evenness of the intestinal microbiota of triploid *O. mykiss* increased. The abundance of the intestinal microbiota reached a significant level when fish were fed 200–800mgkg^−1^ GSH. Results have shown that several species with low microbiotal abundance in the intestines could benefit from dietary GSH supplementation. The change in alpha diversity might be related to the antibacterial properties of GSH, and the mechanism needs further study. In the present study, the predominant intestinal microbiota were Proteobacteria, Firmicutes, and Bacteroidetes, which were similar to those of *O. mykiss* (Huyben et al., 2017; Lyons et al., 2017; Huyben et al., 2021). Some studies have shown that Firmicutes and Bacteroides in the intestine are related to fat deposition, and can ferment more short-chain fatty acids, and promote fat deposition when the proportion of Bacteroides increases (Turnbaugh et al., 2006). This study showed that the abundance of Firmicutes/Bacteroides in each group increased, and the proportion of Actinobacteria with the addition of GSH increased compared with that of the control group. Proteobacteria includes many pathogenic bacteria, such as *Escherichia coli*, *Vibrio cholerae*, and *Salmonella enterica* (Holben et al., 2002), which are usually present in the intestines. In this study, although there were no significant differences in beta diversity among the groups, dietary GSH 200–800mgkg^−1^ increased the abundance of *Ilumatobacter*, *Peptoniphilus*, *Limnobacter*, etc. Moreover, the control diet had an increased abundance of *Arcobacter*, which is responsible for causing diseases in fish. The results in the present study indicated that 200–800mgkg^−1^ dietary GSH may decrease the probability of triploid *O. mykiss* being infected by pathogenic bacteria. This may be related to the role of GSH in scavenging intracellular peroxides and free radicals, protecting cells from oxidative damage. This would maintain the intestinal mucosal barrier, promoting immune cell proliferation, and inhibiting the expression of pro-inflammatory factors, resulting in increased abundance and homogeneity of microbiota in the intestines of triploid *O. mykiss*. However, the microbial functions of the intestine were similar through the functional analysis. Therefore, the dynamics of the microbiota structure and mechanism of action between the altered microbiota and intestine function are still not well understood and need more research.

## Conclusion

In conclusion, dietary GSH is beneficial for triploid *O. mykiss* and may be related to the ability of GSH to scavenge peroxides and free radicals in cells, protect cells from oxidative damage, maintain the intestinal mucosal barrier, promote immune cell proliferation, inhibit the expression of the pro-inflammatory factors, and increase the abundance and evenness of intestinal microbiota. The mechanism of interaction is still unclear, and further research is needed. In addition, more studies are required to elucidate the exact regulatory mechanisms involved by which GSH causes these coordinated responses and explore products (e.g., yeast, wheat germ, and animal liver which contains approximately 1–100mgg^−1^ GSH) or use feed ingredients with high glutathione content appropriately to generate substantial economic returns for the aquaculture industry.

## Data Availability Statement

The datasets presented in this study can be found in online repositories. The names of the repository/repositories and accession number(s) can be found at: https://www.ncbi.nlm.nih.gov/genbank/, PRJNA714809.

## Ethics Statement

The animal study was reviewed and approved by Chinese Animal Health Protection Law and the Scientific Laboratory Animal Permit Approval [Ethical Approval No. SCXK(YU)2005-0001]. Written informed consent was obtained from the owners for the participation of their animals in this study.

## Author Contributions

CW, HL and YY designed the study. BS, YL, and SH carried out the rearing work. CW, BS, HJ, and ZL tested the samples and analyzed the results. CW wrote the manuscript with contributions from the other authors. All authors contributed to the article and approved the submitted version.

## Funding

This study was supported by the Natural Science Funds of Heilongjiang (YQ2019C036), China Agriculture Research System of MOF and MARA (CARS-46), China Scholarship Council (Grant No.202003260012), the Science and Technology Project of Guizhou Province (20162502 and 20162511), the Guizhou Science and Technology Plan Project (QKHZC20172532), the Guizhou Technology Innovation Team Project (QKHRCTD20154016), and the Beijing Sturgeon & Trout Innovation Team (BAIC08-2018).

## Conflict of Interest

The authors declare that the research was conducted in the absence of any commercial or financial relationships that could be construed as a potential conflict of interest.

## Publisher’s Note

All claims expressed in this article are solely those of the authors and do not necessarily represent those of their affiliated organizations, or those of the publisher, the editors and the reviewers. Any product that may be evaluated in this article, or claim that may be made by its manufacturer, is not guaranteed or endorsed by the publisher.

## Footnotes

^1^ http://greengenes.lbl.gov/cgibin/nphindex.cgi
